# Stock Forecasting Based on Informational Complexity Representation: A Framework of Wavelet Entropy, Multiscale Entropy, and Dual-Branch Network

**DOI:** 10.3390/e28040424

**Published:** 2026-04-10

**Authors:** Guisheng Tian, Chengjun Xu, Yiwen Yang

**Affiliations:** 1 School of Economics and Management, Sias University, Xinzheng 451150, China; 10705@sias.edu.cn; 2 School of Artificial Intelligence, Jiangxi Normal University, Nanchang 330022, China; 3 School of Remote Sensing and Information Engineering, Wuhan University, Wuhan 430072, China

**Keywords:** stock price prediction, market state awareness, wavelet entropy, multiscale entropy, cross-modal attention

## Abstract

Stock price sequences are characterized by pronounced nonlinearity, non-stationarity, and multi-scale volatility. They are further influenced by complex, multi-source factors, such as macroeconomic conditions and market behavior, making high-precision forecasting highly challenging. Existing approaches are limited by noise and multi-dimensional market features, as well as difficulties in balancing prediction accuracy with model complexity. To address these challenges, we propose Wavelet Entropy and Cross-Attention Network (WECA-Net), which combines wavelet decomposition with a multimodal cross-attention mechanism. From an information-theoretic perspective, stock price dynamics reflect the time-varying uncertainty and informational complexity of the market. We employ wavelet entropy to quantify the dispersion and uncertainty of energy distribution across frequency bands, and multiscale entropy to measure the scale-dependent complexity and regularity of the time series. These entropy-derived descriptors provide an interpretable prior of “information content” for cross-modal attention fusion, thereby improving robustness and generalization under non-stationary market conditions. Experiments on Chinese stock indices, A-Share, and CSI 300 component stock datasets demonstrate that WECA-Net consistently outperforms mainstream models in Mean Absolute Error (MAE) and R^2^ across all datasets. Notably, on the CSI 300 dataset, WECA-Net achieves an R^2^ of 0.9895, underscoring its strong predictive accuracy and practical applicability. This framework is also well aligned with sensor data fusion and intelligent perception paradigms, offering a robust solution for financial signal processing and real-time market state awareness.

## 1. Introduction

As a cornerstone of contemporary financial systems, stock market price fluctuations not only reflect the prevailing macroeconomic environment but also profoundly influence investment decisions and wealth distribution [1]. Recent studies [2] highlight that global equity markets witness exceptionally high daily trading volumes, where even a marginal reduction of 1% in predictive error could translate into savings of tens of billions of dollars for institutional investors. Notwithstanding the growing significance of accurate forecasting, the inherent complexity of stock prices, stemming from their sensitivity to a multitude of factors such as macroeconomic indicators, corporate fundamentals, and investor sentiment, renders their prediction particularly challenging. These time series exhibit pronounced nonlinearity, non-stationarity, substantial noise, and multiscale patterns [1,3]. The multifaceted and dynamic nature of stock price movements positions their forecasting as one of the most formidable tasks in quantitative finance. Achieving reliable predictions is of practical importance: it aids investors in risk mitigation, assists corporations in refining financial strategies, and supports regulators in safeguarding market stability. Consequently, the advancement of robust predictive models for stock prices has become a focal point in the domains of financial engineering and artificial intelligence.

Stock price forecasting methods are primarily categorized into traditional stock price forecasting, deep learning-based stock price forecasting, and attention mechanism-based stock price forecasting [4,5,6]. Traditional stock price forecasting methods encompass both statistical approaches and conventional machine learning techniques. (1) Traditional statistical forecasting approaches are primarily grounded in time-series theory. By examining properties such as stationarity and autocorrelation in historical price data, classical models such as Autoregressive Integrated Moving Average (ARIMA) and Generalized Autoregressive Conditional Heteroskedasticity (GARCH) have been widely developed and applied [7,8,9]. For instance, Minhaj et al. [10] employed an ARIMA model combined with Johnson & Johnson stock price and S&P 500 index data to construct a forecasting model. Zamzami et al. [11] utilized a symmetric GARCH model to assess IBM stock trading volatility. (2) Traditional machine learning approaches employ nonparametric methods to handle high-dimensional data, primarily including Support Vector Machines (SVMs), Random Forests (RFs), and Artificial Neural Networks (ANNs) [12,13,14]. For instance, Karthick Myilvahanan and Mohana Sundaram [12] proposed an SVM model combined with Long Short-Term Memory (LSTM) and a Convolutional Neural Network (CNN), optimized using the Aquila heuristic optimization algorithm. This approach enabled effective stock market forecasting by processing stock data characterized by high levels of noise, chaotic features, and non-stationarity. Talazadeh and Perakovic [13] enhanced predictive performance by constructing models that combined RF with technical indicators and sentiment analysis. Kan et al. [14] employed an ANN-based stock price forecasting method, leveraging its nonlinear mapping capabilities to process stock data. Although these models can handle nonlinear data, they typically rely heavily on manual feature engineering, and their feature extraction and generalization capabilities remain limited when processing high-dimensional, non-stationary time series data [15,16].

To overcome the limitations of traditional stock price forecasting methods, deep learning techniques have seen growing application in this field. These models can autonomously learn hierarchical and abstract features from raw data, reducing reliance on manual feature engineering and demonstrating robust performance in capturing complex and diverse financial information [17]. Deep learning models such as Recurrent Neural Networks (RNNs), LSTM, and Gated Recurrent Unit (GRU) networks have garnered significant attention due to their ability to effectively capture complex temporal dependencies in time series data [18,19,20]. For instance, Theagarajan et al. [21] employed LSTM, GRU, and RNN models to forecast Indian stock indices. The results demonstrated that these deep learning models can effectively capture intricate market relationships and significantly outperform traditional statistical models, particularly in identifying nonlinear patterns. Xie et al. [22] developed a hybrid forecasting framework that integrates Variational Mode Decomposition (VMD) with Bidirectional Long Short-Term Memory (BiLSTM) to better model the inherent complexity and nonlinearity present in stock market dynamics.

Although deep learning methods are effective at feature extraction and enhancing prediction accuracy, they generally emphasize local patterns and often fail to comprehensively model the long-range global dependencies present in stock markets. To address this limitation, attention-driven approaches have been incorporated into stock price prediction, enabling improved modeling of extended dependencies and complex spatiotemporal structures [23]. For instance, Zena and Al-Sultan [23] proposed an efficient attention-based algorithm for handling noisy and dynamically volatile stock time series data. Singh and Jha [24] combined attention mechanisms with a wavelet denoising LSTM model, employing additive attention to dynamically focus on critical time steps. This approach significantly improved performance in predicting the NIFTY 50 index. Chen et al. [25] developed a stock prediction model integrating variational modal decomposition and multi-scale attention mechanisms to forecast China’s stock market.

Although the aforementioned methods have achieved some success in stock price forecasting, they still exhibit the following limitations:

(1) Most existing approaches fail to perform effective data preprocessing. Stock price data typically exhibits high dimensionality, significant noise, and substantial variability. Existing methods often neglect thorough data cleaning, transformation, and denoising, resulting in models learning features with excessive noise, which compromises prediction accuracy.
(2) Existing approaches predominantly rely on traditional convolutional operations for feature extraction, limiting their ability to capture multi-scale, multi-dimensional market characteristics. Furthermore, these methods often focus solely on price time-series information, while neglecting cross-dimensional correlations between market factors (such as trading behavior, news events, and macroeconomic indicators) and stock prices. This results in incomplete feature extraction and constrained prediction accuracy.
(3) A trade-off exists between prediction accuracy and model complexity in stock forecasting. Achieving high accuracy typically requires complex structural designs, while lightweight models often struggle to deliver satisfactory performance. Existing approaches struggle to strike a balance between prediction precision and computational complexity. This fundamentally stems from the absence of unified, systematic principles in model architecture design, hindering the synergistic optimization of efficient feature representation and computational efficiency.

Stock markets are inherently complex systems characterized by high uncertainty and information asymmetry. Entropy, as a fundamental measure of uncertainty and information content in time series, provides a powerful framework for analyzing the irregularity and complexity of stock price movements. Therefore, this study develops a stock price forecasting model featuring a dual-branch architecture that integrates wavelet decomposition and multimodal attention mechanisms. The main contributions of this work are as follows:

(1) We propose an Informational Complexity Representation framework to characterize the intrinsic complexity of financial time series. This framework integrates wavelet entropy and multiscale entropy as quantitative measures of market information complexity. The wavelet transform is used for multiscale time-frequency representation, and multimodal cross-attention is employed for cross-modal feature fusion. By incorporating entropy-based complexity descriptors, the proposed framework enables the model to better capture the nonlinear dynamics of financial markets and enhance prediction performance.
(2) We design an advanced preprocessing scheme for stock price time series by combining the Yeo–Johnson transformation with a padding-based Fourier transform denoising (P-FTD) method. This approach standardizes data distribution and suppresses noise in raw price records.
(3) We design a multimodal cross-attention mechanism to model the interactions between stock-specific features and macro-market factors. By enabling adaptive fusion of heterogeneous information sources, this mechanism effectively captures the dynamic dependencies between stock prices and macro-market variables. This approach overcomes the limitation of existing models that rely solely on single-stock time-series information. Moreover, the proposed model achieves a balance between prediction accuracy and computational efficiency.

## 2. Related Work

### 2.1. Stock Price Forecasting Models Based on Traditional Methods

#### 2.1.1. Forecasting Methods Based on Traditional Statistics

Traditional statistical forecasting methods are primarily grounded in time-series theory. By examining properties such as stationarity and autocorrelation in historical price data, several classical models have been developed. Among them, the ARIMA [26] model is widely used for predicting price levels, whereas the GARCH [27] model is primarily employed for modeling and forecasting volatility (variance). In this study, we adopt ARIMA as the statistical baseline for price level prediction and use it as a baseline for fair comparison with other methods. For instance, Cao [28] employed an ARIMA model to forecast Xiaomi Auto’s stock price over the next 30 days after conducting stationarity tests and differencing. Singh et al. [29] utilized multiple GARCH variants for stock return volatility forecasting and found that the Exponential Generalized Autoregressive Conditional Heteroskedasticity (EGARCH) model demonstrated higher accuracy. Salisu et al. [30] employed GARCH to process mixed-frequency data and confirmed that action-based geopolitical risk indices possess significant predictive power for emerging market stock volatility. Nevertheless, such models are mainly confined to modeling linear relationships and perform poorly when confronted with the nonlinear dynamics of financial markets [31].

#### 2.1.2. Prediction Methods Based on Traditional Machine Learning

As computational capabilities and data availability continue to grow, machine learning techniques have become increasingly important in financial forecasting. Unlike traditional statistical approaches, these methods are capable of processing high-dimensional and nonlinear datasets without stringent assumptions about data distribution. For instance, Bhuyan et al. [32] compared the performance of decision trees, RFs, and SVMs for predicting stock prices using insider trading data. Mallam et al. [33] noted thatANNs and RF demonstrated strong performance in multiple company closing price prediction tasks, as measured by root mean square error (RMSE) and mean absolute percentage error (MAPE). Ahmed et al. [34] also highlighted that models such as ANNs are adept at identifying nonlinear patterns in stock price fluctuations and capturing intricate interactions among variables. Although these models can handle nonlinear data to some extent, they typically require specialized domain knowledge, and their ability to extract informative features and generalize effectively can be limited when applied to high-dimensional and non-stationary financial time series data [35]. Additionally, these models are often susceptible to noise and overfitting and typically offer limited interpretability [36].

### 2.2. Deep Learning-Based Stock Price Prediction Models

#### 2.2.1. Single Deep Learning Models

Single deep learning approaches typically utilize specific architectures to process stock price data, such as LSTM [37], RNN [38], or CNN [39]. For instance, Huang et al. [40] utilized LSTM for stock price forecasting and combined it with an evolutionary weighting strategy to develop trading strategies. Hossain et al. [41] noted that GRUs reduce computational complexity while maintaining performance comparable to other recurrent models. Cao [39] pointed out that CNNs can capture localized patterns in time-series data through one-dimensional convolution operations. In stock forecasting, this enables the identification of “shapes” or local trends within stock price charts, which provide crucial clues for prediction.

#### 2.2.2. Hybrid Deep Learning Models

To enhance robustness and generalization capabilities, numerous researchers have combined distinct deep learning models into hybrid architectures, making this approach the mainstream method for stock price forecasting. For instance, Singh et al. [42] proposed a hybrid model integrating CNNs and LSTMs for stock price prediction and demonstrated outstanding performance in stock price time series forecasting. Boddu and Manimaran [43] introduced a hybrid approach integrating Time Variance Embedding (TVE) and Multi-Scale Convolutional Blocks to enhance stock price forecasting performance. Liu et al. [44] introduced an LSTM-based framework that incorporates multi-scale convolutional feature fusion, enabling it to better cope with the inherent randomness and complexity of stock market dynamics. Lin et al. [45] developed a hybrid framework combining Ensemble Empirical Mode Decomposition (EEMD), CNN, and an attention-augmented LSTM, which can disentangle volatility components in stock prices and emphasize critical trend variations. Their research indicates that the mean prediction error decreased by over 15% compared to traditional time series frameworks. Bongale et al. [46] proposed a hybrid LSTM model incorporating social media sentiment from blended datasets, demonstrating that external information enhances prediction accuracy. Joshi et al. [47] further explored the application of multi-agent LLM frameworks for financial feature summarization and stock recommendation, representing a cutting-edge direction in integrating large language models with financial decision-making. Additionally, Dharrao et al. [48] investigated the application of models such as BART and T5 in financial news summary generation, offering novel insights for effective extraction of financial textual information.

### 2.3. Attention-Based Stock Price Forecasting Models

Because deep learning-based stock price forecasting models often struggle to capture long-range dependencies—potentially resulting in lower prediction accuracy—scholars have proposed attention-based deep learning models. Unlike traditional neural networks, attention-based models can dynamically focus on components that significantly influence prediction outcomes at different time steps, thereby enhancing performance. For instance, Li and Xu [49] combined generative adversarial networks (GANs) with Transformer-based attention to better capture long-term dependencies in financial time series, thereby improving prediction accuracy over traditional CNN-based approaches. Burak Gülmez [50] proposed GA-Attention-Fuzzy-Stock-Net, which integrates genetic algorithms, attention mechanisms, and neural fuzzy systems for stock price forecasting. Arul Goutham et al. [51] employed VMD to decompose stock data into multiple modalities and combined attention mechanisms with LSTM network training to improve intraday stock price prediction accuracy. Although attention mechanisms enhance global feature modeling capabilities, existing research still leaves room for improvement in multi-scale feature fusion and noise suppression. This gap motivates the development of hybrid models that combine multi-resolution wavelet transforms, CNNs, and attention mechanisms.

## 3. Method

### 3.1. Model Overview

**Figure 1:**
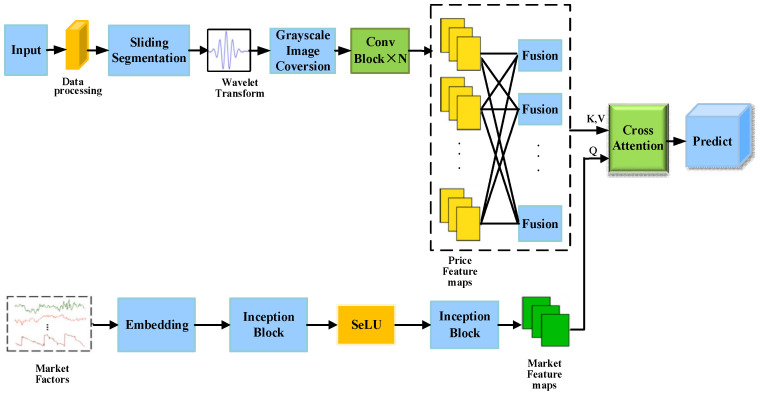
Overall Model Architecture Diagram.

This section details the proposed WECA-Net (Wavelet Entropy and Cross-Attention Network) model, as illustrated in Figure 1. The model adopts a dual-branch architecture. The upper branch processes stock price sequences by converting one-dimensional data into two-dimensional spatiotemporal maps via wavelet transforms, then extracts multi-scale spatiotemporal features using multi-level convolutional modules. The lower branch processes macroeconomic market factors, extracting multi-scale dynamic features through an enhanced Inception module. Features from both branches are fused through a cross-attention mechanism, where market factor features serve as queries that guide the model to focus on price features most relevant to the current market state. Additionally, the model explicitly computes wavelet entropy and multiscale entropy as quantitative representations of information complexity, which are concatenated with the extracted features before being input to the fusion module. This integrated design aims to simultaneously capture multiscale volatility patterns within prices and the influence of external market conditions, thereby enhancing prediction accuracy and robustness.

### 3.2. Data Preprocessing

Since stock market data contains noisy or anomalous values that may impact subsequent learning, comprehensive data preprocessing is required before feeding raw stock data into deep learning models. This process primarily involves the following two steps.

#### 3.2.1. Yeo–Johnson Transformation

Stock price time series typically exhibit significant heteroscedasticity and non-normal distribution characteristics, which severely impact the stability of subsequent deep learning model training and prediction accuracy [52]. To address this issue, we first apply the Yeo–Johnson transformation to the raw stock price sequence ($P=\left\{p_{1},p_{2},\ldots ,p_{T}\right\}$). This transformation is a power transformation technique whose main advantage lies in stabilizing the variance of the sequence and making the data distribution more closely approximate normality. The resulting transformed features are more regular, which not only facilitates optimization for deep learning models but also enhances robustness to outliers, thereby laying a solid foundation for subsequent accurate predictions. For each data point $p_{t}$, the transformation function is defined as:

To address this issue, we first apply the Yeo–Johnson transformation to the raw stock price sequence $P=\left\{p_{1},p_{2},\ldots ,p_{T}\right\}$. This transformation is a power transformation method whose core advantage lies in stabilizing the variance of the sequence and making the data distribution closer to normal. The transformed data features become more regular, not only facilitating optimization by deep learning models but also enhancing robustness against outliers, thereby laying a solid foundation for subsequent accurate predictions. For each data point $p_{t}$, the transformation function is defined as:

(1) $$\psi_{\lambda }\left(p_{t}\right)=\left\{\begin{array}{c} \frac{{\left(1+p_{t}\right)}^{\lambda }-1}{\lambda }\;\;\;\;\;\;\;\;\;\;\;\;\;\;\;\;\;\;\;\;\;\;\;\mathrm{if}\;\lambda \neq 0\;\mathrm{and}\;p_{t}\geq 0\\ \ln\left(p_{t}+1\right)\;\;\;\;\;\;\;\;\;\;\;\;\;\;\;\;\;\;\;\;\;\;\;\;\;\;\;\;\;\mathrm{if}\;\lambda =0\;\mathrm{and}\;p_{t}\geq 0\\ \frac{{-\{\left(-p_{t}+1\right)}^{2-\lambda }-1\}}{2-\lambda }\;\;\;\;\;\;\;\;\;\;\mathrm{if}\;\lambda \neq 2\;\mathrm{and}\;p_{t}<0\\ -\ln\left(-p_{t}+1\right)\;\;\;\;\;\;\;\;\;\;\;\;\;\;\;\;\;\;\;\;\;\;\mathrm{if}\;\lambda =2\;\mathrm{and}\;p_{t}<0 \end{array}\right.$$

where $p_{t}$ denotes the original price sequence data point, λ represents the optimal transformation parameter determined via maximum likelihood estimation, $\psi_{\lambda }\left(p_{t}\right)$ is the transformed data point, and the transformed sequence is denoted as $X=\left\{x_{1},x_{2},\ldots ,x_{T}\right\}$. This transformation simultaneously handles positive and negative values and improves the distributional properties of the stock price sequence. It optimizes transformation effectiveness through a data-driven approach, thereby significantly enhancing the quality of input data and the overall adaptability of the model.

#### 3.2.2. P-FTD Denoising

After stabilizing the variance via the Yeo–Johnson transformation, we further introduce the P-FTD model to eliminate residual noise [53]. P-FTD is an advanced signal denoising technique whose core principle is to distinguish and remove noise components using adaptive threshold filtering in the frequency domain. The core process of this model is described in Algorithm 1 as follows:
**Algorithm 1.** P-FTD Denoising AlgorithmInput: Transformed sequence $X=\left[x_{1},x_{2},\cdots \;,x_{T}\right]$ Padding length p=10 Padding distribution hyperparameter α = 0.5 Output: Denoised sequence $\tilde{X}=\left\{{\tilde{x}}_{1},{\tilde{x}}_{2},\ldots ,{\tilde{x}}_{T}\right\}$ 1. Compute the standard deviation σ of the sequence X. 2. Generate the starting padding sequence: $\varepsilon_{i}\sim N\left(0,{\alpha \sigma }^{2}\right),i=1,\cdots ,p$
3. Generate the ending padding sequence: $\varepsilon_{P+i}\sim N\left(0,{\alpha \sigma }^{2}\right),i=1,\cdots ,p$ 4. Construct the padded sequence:
$X^{pad}=\left\{\varepsilon_{1},\cdots ,\varepsilon_{p},x_{1},\cdots ,x_{T},\varepsilon_{P+1},\cdots ,\varepsilon_{2P}\right\}$ 5. Perform Fast Fourier Transform:$\;F_{fft}=FFT\left(X^{pad}\right)$ 6. Compute the frequency domain threshold: $\tau_{f}=P_{90}\left(\left|F_{k}\right|\right)$//where $P_{90}$ denotes the 90th percentile of the magnitudes of the frequency components. 7. For each frequency component $F_{k}$, apply filtering:        If $\left|F_{k}\right|<\tau_{f}$, set $F_{k}=0$        Otherwise, keep $F_{k}$ unchanged. 8. Perform inverse Fourier transform: $X^{filtered}=IFFT\left(F_{fft}\right)$ 9. Remove the padding data from both ends to obtain the denoised sequence
$\tilde{X}$.

P-FTD employs a data-driven dynamic thresholding strategy that effectively eliminates high-frequency noise while preserving valid signal components representing genuine market fluctuations, maximizing the retention of low-frequency valid signals reflecting authentic market volatility [53]. Following P-FTD processing, we obtain a smoother and cleaner stock price sequence dataset $\tilde{X}=\left\{{\tilde{x}}_{1},{\tilde{x}}_{2},\ldots ,{\tilde{x}}_{T}\right\}$, providing high-quality input for subsequent time-frequency analysis modules.

To assess the effect of preprocessing on stationarity, we performed Augmented Dickey–Fuller (ADF) unit root tests on both the raw price series and the preprocessed series. The results (see Table 1) show that the raw series is non-stationary at the 5% level, while the series after Yeo–Johnson transformation and P-FTD denoising becomes stationary at the 1% level. This ensures that subsequent prediction modeling avoids spurious regression due to unit roots.

We conducted ADF unit root tests on all financial instruments used in this study. For illustration purposes, Table 1 reports the results for a representative subset (3 indices, 3 A-shares, 3 CSI 300 constituents). The lag length was automatically selected based on the Akaike Information Criterion (AIC), with a maximum lag set to $\mathrm{floor}\left(12\times (T/100{)}^{1/4}\right)$, where T=1457. Significance levels are reported at 1%, 5%, and 10%. For the raw price series, all *p*-values exceed 0.05, indicating that the null hypothesis of a unit root cannot be rejected. This confirms that the raw price series are non-stationary, consistent with the typical random walk behavior of financial time series. After the Yeo–Johnson transformation and P-FTD denoising, all *p*-values are below 0.01, allowing rejection of the unit root at the 1% level. This demonstrates that the preprocessed series becomes stationary, providing a valid statistical foundation for level prediction and mitigating concerns about spurious regression.

It is crucial to emphasize that all P-FTD parameters (padding length, scaling factor, and frequency-domain threshold) are estimated solely from the training set. During validation and testing, for each time step to be predicted, we apply denoising only to the historical sequence available up to that point (i.e., all data prior to that time). The last window is then extracted from the denoised sequence as input. This sequential procedure guarantees that no future information is ever used, eliminating look-ahead bias entirely. Consequently, the test data are not processed by a “global” filter in a single pass, but are denoised in a rolling real-time manner, ensuring statistical validity.

### 3.3. Stock Price Data Segmentation

**Figure 2:**
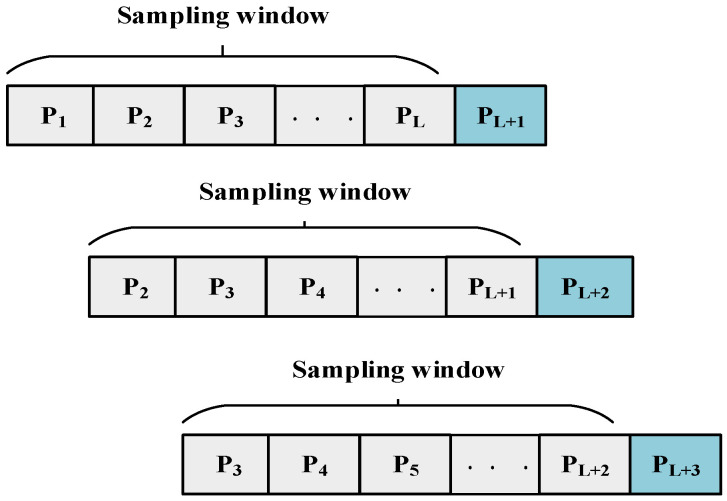
Dividing Stock Price Data Using a Sliding Window.

To transform continuous financial time series into supervised learning samples suitable for deep learning models, this study employs a sliding window segmentation method to structure the preprocessed price sequence $\tilde{X}$ [54]. The core objective of this approach is to establish explicit mappings between historical price patterns and future trends, thus providing the model with training samples that capture temporal dependencies. The detailed processing workflow is illustrated in Figure 2.

Based on the temporal characteristics of time series, three key parameters are first defined. The window length L determines the historical observation period, the forecast step F specifies the future prediction horizon, and the sliding step ∆ controls the sampling density. In this study, the forecasting task is defined as one-step forecasting, and the forecasting step size is set to F=1. The segmentation process begins at the sequence start point, sequentially extracting price data from consecutive L time points to form the input feature sequence:

(2) $$S_{i}=\left\{{\tilde{x}}_{t},{\tilde{x}}_{t+1},\ldots ,{\tilde{x}}_{t+L-1}\right\}$$

The prediction target is defined as the price value at the first time point after the window closes:

(3) $$Y_{i}={\tilde{x}}_{t+L}$$

For a denoised sequence of length T, sliding with step ∆ yields

(4) $$N=\left\lfloor \frac{T-L}{\Delta }\right\rfloor +1$$

sample pairs $\left(S_{i},Y_{i}\right)$, i=1,2,⋯,N, where each $S_{i}$ is a historical stock price sub-sequence of length L, and $Y_{I}$ is its corresponding future price target. This standardized data processing approach provides a normalized input format for subsequent wavelet transform feature extraction. It ensures a smooth transition from one-dimensional time-series data to two-dimensional time-frequency images and establishes a high-quality training foundation for deep learning models.

### 3.4. Wavelet Transform and Grayscale Conversion

To quantify the complexity of market information, this paper introduces two entropy-based measures: wavelet entropy and multiscale entropy. Wavelet entropy captures the uncertainty in the distribution of energy across different frequency components of stock price sequences. When energy is concentrated in a limited number of frequencies, the entropy value is low, indicating strong periodicity or regularity. Conversely, if energy is spread across a wide range of frequencies, the entropy value is high, reflecting greater complexity and unpredictability. Multiscale entropy, on the other hand, assesses complexity by examining the self-similarity and regularity of a sequence across multiple time scales. The variation in entropy values with scale reveals whether the sequence exhibits long-range correlations or tends toward randomness. Integrating these entropy features into predictive models enhances their ability to perceive dynamic market shifts, thereby improving prediction robustness.

**Figure 3:**
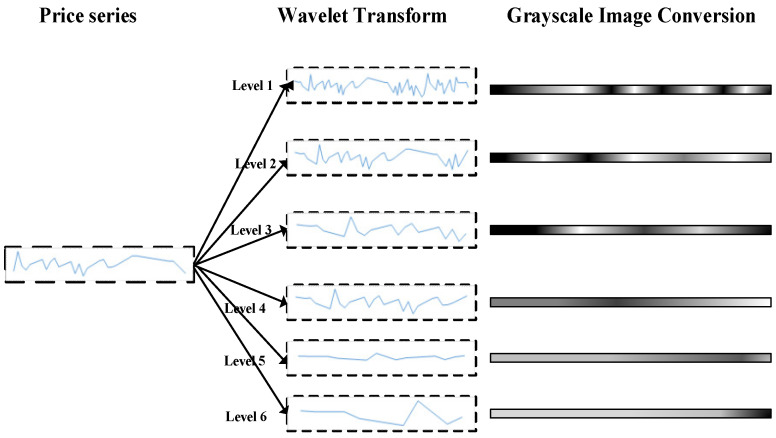
Wavelet Transform and Grayscale Conversion Diagram.

For the subsequence samples obtained via sliding window segmentation, this study employs multi-scale wavelet decomposition combined with grayscale conversion to transform one-dimensional time series into two-dimensional time–frequency image representations. This approach enhances the model’s capacity to capture multi-scale dynamic patterns inherent in financial time series [2]. The transformation not only reveals localized fluctuations of price sequences across different frequency scales but also provides a structured input format conducive to subsequent convolutional feature extraction. The overall procedure is formalized in Algorithm 2, and an illustrative example of the resulting data representation is shown in Figure 3. It is worth noting that Algorithm 2 and Figure 3 specifically depict the generation of time–frequency images based on the Continuous Wavelet Transform (CWT), which serves as the input representation for the convolutional neural network. In contrast, the entropy feature is a quantitative measure of complexity derived from Discrete Wavelet Transform (DWT) coefficients using a defined formula. Together, these two components constitute a multi-perspective characterization of market information complexity within the proposed model.

In Algorithm 2, the scale vector $a=[a_{1},a_{2},\ldots ,a_{M}]$ represents the multiscale parameters in continuous wavelet analysis. Although this notation originates from the theoretical definition of the continuous wavelet transform, this paper employs a discrete implementation for practical computations. Multiscale convolution calculations are performed by constructing corresponding wavelet filters at different scales $a_{M}$.
**Algorithm 2.** Haar Wavelet-Based Time-Frequency Spectrum Image Generation AlgorithmInput: $S=\left\{X_{1},X_{2},\cdots ,X_{N}\right\}$: Sample set, where each sample $X_{i}=\left[{\tilde{x}}_{1},{\tilde{x}}_{2},\cdots \;,{\tilde{x}}_{L}\right]$ is a sub-sequence of length L, denoised via P-FTD and segmented by sliding window wavelet_base_: Selected mother wavelet function (Haar wavelet used in this study) scales: The scale vector a to be analyzed: $a=[a_{1},a_{2},\cdots \;,a_{M}]$ img_height_, img_width_: Desired output size of the time-frequency image (H×W) (Fixed at 64×64 in the experiment) Output: $I=\left\{I_{1},I_{2},\cdots ,I_{N}\right\}$: Set of time-frequency images, where each $I_{i}$ is a grayscale image of size H×W 1. I←{} // Initialize empty set 2. // Generate wavelet filters for all scales based on the mother wavelet and scale vector 3. filters←generate_wavelet_filters (wavelet_base_, *a*) // The function constructs a bank of wavelet filters, one for each scale $a_{m}$. 4. for i←1 to N do 5.           $C\leftarrow 0^{M\times L}$ // Initialize wavelet coefficient matrix 6.         for m←1 to M do 7.              Convolve $X_{i}$ with the filter filters[m] using convolution mode ‘same’ to obtain coefficient vector $c_{m}$ // ‘same’ mode ensures the output length equals the input length L. 8.            Store the result: C[*m*,:]←cm 9.         end for 10. Compute power spectrum matrix: $P\leftarrow {\left|C\right|}^{2}$//Element-wise square of magnitudes; $P\in R^{M\times L}$. 11. Normalize to $\left[0,1\right]$: $P_{norm}\leftarrow \frac{P-min(P)}{max(P)-min(P)}$ 12. Resize $P_{\mathrm{norm}}$ to fixed size H×W using bilinear interpolation: $I_{i}=\mathrm{resize}(P_{\mathrm{norm}},H,W)$ 13. Add image to set: $I\leftarrow I\cup \{I_{i}\}$ 14. end for 15. return I


#### 3.4.1. Construction of the Wavelet Time-Frequency Representation

For any subsequence $S=\left\{{\tilde{x}}_{1},{\tilde{x}}_{2},\cdots ,{\tilde{x}}_{L}\right\}$ (for brevity, subscripts start from 1), this paper employs Haar wavelets for four-level discrete wavelet decomposition. The decomposition produces detail coefficients at multiple scales $D_{j}\left(j=1,2,3,4\right)$ and an approximation coefficient sequence representing the low-frequency component of the signal $A_{4}$. Detail coefficients $D_{j}$ capture local fluctuation characteristics of the price sequence at different frequency scales, where the subscript j denotes the decomposition level (i.e., scale). The length of the coefficient sequence at each scale corresponds to the number of sample points at that scale, with the coefficient position indicated by the index k. The approximation coefficient $A_{4}$ reflects low-frequency trend information, also indexed by k. This multiresolution decomposition simultaneously preserves information in both the time and frequency domains, enabling clear expression of abrupt changes, periodic fluctuations, and long-term trends across various scales, thereby providing a foundation for complex pattern analysis.

By stacking scale coefficients vertically according to frequency scales and horizontally according to temporal evolution, a two-dimensional time-frequency matrix can be constructed. This matrix reflects scale variations vertically and temporal evolution horizontally, thereby forming a two-dimensional representation describing the dynamic structure of prices.

#### 3.4.2. Calculation of Information Complexity Entropy Features

To further characterize the information complexity of the sequence, this paper calculates two types of entropy features based on the wavelet decomposition results.

(1) Wavelet Entropy

Wavelet entropy is used to quantify the distribution of signal energy across different wavelet scales, thereby characterizing the information complexity of financial time series. First, calculate the energy distribution of coefficients at each scale. The energy at the jth detail scale is defined as:

(5) $$E_{j}=\sum_{k}{\left|D_{j,k}\right|}^{2},j=1,\cdots ,4$$

where $D_{j,k}$ denotes the wavelet detail coefficient at the kth position in the jth scale. The energy of the approximation component is defined as:

(6) $$E_{5}=\sum_{k}{\left|A_{4,k}\right|}^{2}$$

Here, $A_{4,k}$ denotes the approximation coefficient at the kth position. The total sequence energy is:

(7) $$E_{tot}=\sum_{j=1}^{5}E_{j}$$

This yields the normalized probability for energies at each scale:

(8) $$p_{j}=\frac{E_{j}}{E_{tot}}$$

Based on the Shannon entropy formulation, the wavelet entropy is defined as:

(9) $$H_{w}=-\sum_{j=1}^{5}p_{j}\log p_{j}$$

This metric measures the uncertainty in energy distribution across the frequency domain of price sequences: lower entropy indicates energy concentration at fewer scales, while higher entropy reflects uniform distribution. Thus, wavelet entropy reflects the frequency-domain complexity of market volatility.

(2) Multiscale Entropy

To characterize the structural features of a sequence at different temporal resolutions, this paper introduces a multiscale entropy approach. For the denoised complete price sequence, coarse-graining is first applied. At scale factor s, the coarse-grained sequence is defined as:

(10) $$y_{k}^{\left(s\right)}=\frac{1}{s}\sum_{i=\left(k-1\right)s+1}^{ks}{\tilde{x}}_{i},k=1,2,\cdots ,\left\lfloor \frac{T}{s}\right\rfloor$$

where s=1,2,⋯,10 denotes the scaling factor, k represents the time index after coarsening. Subsequently, the sample entropy of each coarsened sequence is computed:

(11) $$S\left(s\right)=SampEN\left(m,r,y^{\left(s\right)}\right)$$

Here, the embedding dimension is set to m=2, and the tolerance is set to $r=0.2\times \sigma_{\tilde{x}}$, where $\sigma_{\tilde{x}}$ is the standard deviation of the original denoised sequence $\tilde{X}$. The sample entropy value reflects the self-similarity and regularity of the sequence at different scales. When the scale factor s=1,2,⋯,10, the multiscale entropy vector is obtained:

(12) $$E_{MSE}=\left[S\left(1\right),S\left(2\right),\cdots ,S\left(10\right)\right]$$

This vector describes the complex structure of price sequences across different time scales: high entropy values indicate stronger randomness in the sequence, while low entropy values reflect the presence of distinct structural patterns.

In the subsequent feature fusion stage, wavelet entropy $H_{w}$ and multiscale entropy vector $E_{MSE}$ serve as information complexity features. Together with time-frequency features extracted by convolutional neural networks and market factor features, they participate in model learning, thereby enhancing the model’s ability to capture market uncertainty and complex dynamics.

#### 3.4.3. Grayscale Image Generation

After obtaining the multiscale wavelet coefficients for each sub-sequence S, the multiscale time-frequency information is mapped into a two-dimensional matrix by calculating the energy spectrum of coefficients at each scale and performing normalization. Specifically, for each scale j and time position k, the energy value is derived from the square of the wavelet coefficient ${\left|D_{j,k}\right|}^{2}$ (for detail coefficients) or ${\left|A_{4,K}\right|}^{2}$ (for approximation coefficients). Stack the energies from all scales row-wise to form an initial two-dimensional energy matrix. Its number of rows equals the number of decomposition scales, and its number of columns equals the maximum length of coefficients at each scale. Since wavelet decomposition yields coefficients of varying lengths at different scales, interpolation (e.g., bilinear interpolation) is required to uniformly adjust the length of each row to the predetermined number of columns. Subsequently, the matrix undergoes normalization, mapping energy values to the interval [0, 1]. A scale transformation then scales the matrix to a fixed size H×W, ultimately generating a single-channel grayscale image where each pixel’s grayscale value corresponds to the normalized energy intensity. This image not only preserves the multi-scale spatiotemporal features of the original time series but also highlights key structural information within price fluctuations. It provides convolutional neural networks with an input format that aligns with their spatial perception mechanisms. The generated spatiotemporal grayscale image is then fed into a feature extraction module to further uncover higher-order spatial features, enabling deep modeling of complex dynamic patterns within financial time series.

### 3.5. Feature Extraction Module

To efficiently and robustly extract multi-level features from the aforementioned wavelet time-frequency images, we abandon the traditional convolutional stacking approach. Instead, we adopt a modern architecture that integrates DWConv, Squeeze-and-Excitation (SE), Scaled Exponential Linear Unit (SeLU) activation functions, and dual residual connections. This architecture is stacked N times to form the core feature extraction backbone network, designed to achieve richer and more robust feature representations at a lower computational cost. As the central feature extraction unit in the stock price branch, this module forms a counterpart to the market factor branch at the structural level, jointly establishing the dual-channel feature representation foundation of the model.

**Figure 4:**
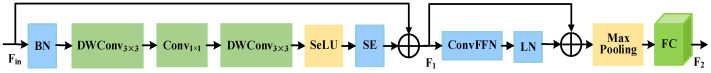
Multi-Feature Extraction Module.

The structure of the feature extraction module is illustrated in Figure 4.

The formula is as follows:

(13) $$F_{1}=SE(SeLU\left({DWConv}_{3\times 3}\left({Conv}_{1\times 1}({DWConv}_{3\times 3}\left(BN\left(F_{in}\right)\right)\right)\right)))+F_{in}$$

(14) $$F_{2}=FC\left(MaxPool\left(LN\left(ConvFFN\left(F_{1}\right)\right)+F_{1}\right)\right)$$

where $F_{in}\in R^{H\times W}$ denotes the input feature map, BN ($F_{in}$) represents batch normalization, DWConv_3×3_ denotes depthwise separable convolution with a 3 × 3 kernel, Conv_1×1_ denotes a standard 1 × 1 convolution kernel, SeLU(·) represents the self-normalizing activation function, SE(·) denotes the Squeeze and Excitation (SE) attention module, +$F_{in}$ indicates the first residual connection, ConvFFN(F_1_) signifies the convolutional feedforward network, LN(·) denotes layer normalization, and +F_1_ represents the second residual connection.

Specifically, feature maps from data samples first undergo batch normalization (BN) to accelerate model convergence [55,56]. Subsequently, a 3 × 3 depthwise separable convolution extracts local spatiotemporal patterns in the spatial dimension, significantly reducing computational complexity. Following this, a 1 × 1 standard convolution (Conv_1×1_) performs inter-channel feature fusion and dimensionality transformation to enhance feature expressiveness. Data undergoes further spatial feature refinement via 3 × 3 depthwise separable convolution, incorporating self-normalizing nonlinear transformation through the SeLU activation function to stabilize gradients. The SE module then aggregates global channel information using global average pooling and determines the significance of each channel through a fully connected layer, which allows for adaptive adjustment of feature channels. Next, the recalibrated features are integrated with the original input $F_{in}$ via an initial residual connection to retain essential low-level information. The resulting features are subsequently passed into a convolutional feedforward network (ConvFFN) [56], where two 1 × 1 convolutional layers enhance nonlinear representation capabilities, followed by layer normalization (LN) to stabilize the training process. Finally, a second residual connection fuses the LN output with the first residual result, enhancing feature reuse. Through deep convolution, channel attention, dual residual connections, and feedforward networks, the entire multi-feature extraction module precisely extracts key patterns from spatiotemporal images, improving stock price prediction accuracy.

### 3.6. Market Feature Extraction Module

**Figure 5:**
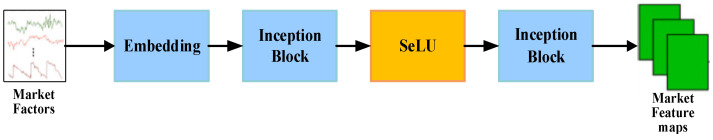
Market Factors Module.

In addition to the stock’s own price data, stock prices are also closely linked to broader market conditions and macroeconomic factors [25]. To capture the potential impact of these external factors on stock prices, we designed a dedicated market factors branch, as shown in Figure 5.

The input to this module is multidimensional market factor time series data $M\in R^{T\times D}$, where T represents the time step (aligned with the stock price sequence), and D denotes the market factor dimension. The specific processing flow is as follows: First, the input market factors undergo embedding to obtain relevant features. Then, the Inception module performs multi-scale feature extraction while applying the SeLU activation function for nonlinear transformation. Subsequently, the Inception module conducts deep abstraction to extract higher-order feature interaction patterns. Ultimately, this branch outputs high-quality market factor features $F_{\mathrm{market}}$, which serves as the query vector (Query) fed into the subsequent dual attention fusion module. Here, they intelligently interact with historical price features to jointly accomplish the stock price prediction task.

**Figure 6:**
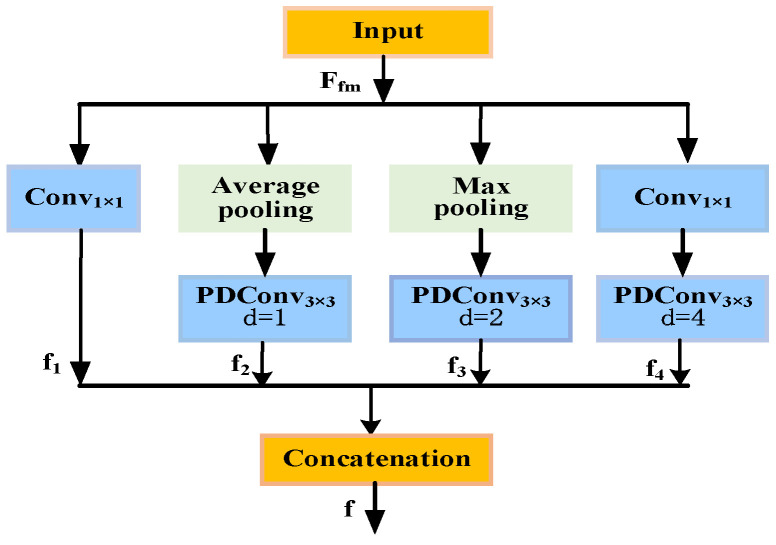
Inception Block.

The Inception Block primarily performs convolutions and pooling with varying receptive fields to capture diverse feature interaction patterns from micro to macro scales. As illustrated in Figure 6, this module comprises four parallel branches: the first branch performs 1 × 1 convolutions for basic feature projection; the second branch first extracts relevant features via average pooling, then applies parallel dilated convolutions with 3 × 3 kernels and dilation rate d = 1 to capture smoothed local features; the third branch performs max pooling followed by parallel dilated convolutions with a 3 × 3 kernel and dilation rate d = 2 to capture prominent medium-range features; the fourth branch applies a 1 × 1 dilated convolution followed by parallel dilated convolutions with a 3 × 3 kernel and dilation rate d = 4 to obtain global macro-level features. Features from these four branches are fused via element-wise summation.

The formula is as follows:

(15) $$f_{1}={Conv}_{1\times 1}\left(F_{mf}\right)$$

(16) $$f_{2}={PDConv}_{3\times 3,d=1}\left(AvgPool\left(F_{mf}\right)\right)$$

(17) $$f_{3}={PDConv}_{3\times 3,d=2}\left(MaxPool\left(F_{mf}\right)\right)$$

(18) $$f_{4}={PDConv}_{3\times 3,d=4}\left({Conv}_{1\times 1}\left(F_{mf}\right)\right)$$

(19) $$f=f_{1}+f_{2}+f_{3}+f_{4}\;\;\;\;$$

where $F_{mf}$ denotes the market factor input tensor, Conv_1×1_(⋅) denotes a 1 × 1 convolution operation, used to achieve channel dimension mapping and feature compression, thereby reducing redundancy and enhancing computational efficiency; AvgPool(⋅) and MaxPool(⋅) represent average pooling and max pooling operations, respectively, used to extract global trend information and significant local response features of market factors from different statistical perspectives; PDConv_3×3, d = k_(⋅) denotes a 3 × 3 padding-based dilated convolution with dilation rate k. This operation expands the receptive field while maintaining a manageable parameter scale, enabling capture of market dynamics across different scales. f_1_–f_4_ denote multi-scale feature representations extracted from different branches. These are ultimately fused through element-wise summation to yield the comprehensive multi-scale market factor feature representation f, providing high-quality input for subsequent cross-attention mechanisms.

This multi-branch architecture enables the model to learn hierarchical representations step-by-step, from basic statistical features to advanced semantic features. It fully explores the latent and complex nonlinear relationships between market factors and stock price movements, providing high-quality feature inputs for the subsequent multimodal cross-attention mechanism. Simultaneously, in terms of network depth and feature abstraction level, the market factor branch maintains a symmetrical design with the stock price feature branch. This ensures relatively balanced modeling capabilities for different information sources within a unified structural framework, thereby enhancing the stability of multi-source information fusion and overall prediction performance.

### 3.7. Multimodal Cross-Attention Mechanism Integrating Market Factor Features and Stock Price Features

**Figure 7:**
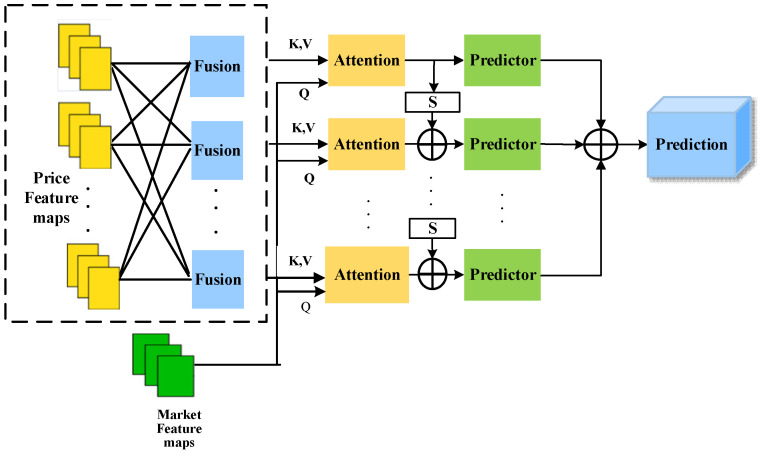
Cross-Attention Mechanism Module.

When integrating historical price features with market factor features, simple concatenation or weighted averaging methods struggle to adapt to complex market dynamics. To address this, this paper designs a multimodal cross-attention mechanism to effectively fuse historical stock price features with market factor features. In this mechanism, market factor features serve as query signals, guiding the model to dynamically focus on information within stock price features that is most relevant to the current market state. In this way, adaptive fusion of multi-source features is achieved. Constrained by an overall dual-branch structure, the mechanism balances flexibility in information interaction with structural stability, as shown in Figure 7.

This module fuses stock historical price features extracted at different time scales by the upper branch, forming unified price feature maps $F_{\mathrm{price}}$, which serve as both keys and values in the attention mechanism. Simultaneously, the multi-scale market factor feature maps $F_{\mathrm{market}}$ extracted by the lower branch serve as queries.

Additionally, the wavelet entropy $H_{w}$ and multiscale entropy vector $E_{MSE}$ computed in Section 3.4 are regarded as representations of information complexity. These entropy features characterize the uncertainty and structural complexity of stock price sequences across different frequency and time scales. To fully leverage this information, this paper concatenates entropy features with price features, forming an extended price feature representation that jointly participates in subsequent attention computations. Through this approach, the attention mechanism not only focuses on the time-frequency structure of price sequences but also references changes in market information complexity, thereby more effectively identifying historical patterns with significant predictive significance.

Through this cross-attention modeling approach based on market state queries, the model dynamically retrieves and integrates the most relevant information from historical price patterns guided by the current market environment. This enables more effective characterization of market factors’ influence on stock price movements. For the nth pair of stock price features and market-related features, the corresponding attention weight is computed as follows:

(20) $$Q_{n}=F_{\mathrm{market}}^{\left(n\right)}W_{q}\;$$

(21) $$K_{n}=F_{\mathrm{price}}^{\left(n\right)}W_{k}$$

(22) $$V_{n}=F_{\mathrm{price}}^{\left(n\right)}W_{v}$$

(23) $${\mathrm{Attn}}_{n}=\mathrm{softmax}\left(\frac{Q_{n}K_{n}^{⊤}}{\sqrt{d_{k}}}\right)V_{n}$$

where $n\in \left[1,N\right]$, $W_{q}$, $W_{k}$, and $W_{v}$ are weight matrices, $\sqrt{d_{k}}$ represents the scaling factor, which prevents gradient vanishing caused by excessively large dot products. This attention mechanism effectively integrates market factors and stock price sequence information, capturing and understanding their interactions while highlighting critical time points within the stock price sequence to preserve more valuable insights.

To enhance the robustness and effectiveness of feature fusion, a gated recurrent optimization mechanism is incorporated. This mechanism iteratively optimizes the attention outputs by combining a forget gate (Sigmoid) and an input gate (Tanh). Under this approach, the model adaptively modulates the information flow across features at multiple temporal scales, emphasizing salient patterns while attenuating noise and irrelevant components.

The attention outputs are subsequently fed into the prediction layer to produce intermediate forecasts for each sub-window. The final prediction $\hat{y}$ is obtained by aggregating the outputs across all N sub-windows:

(24) $$\hat{y}=\sum_{n=1}^{N}{\mathrm{Predictor}}_{n}\left({\mathrm{Attn}}_{n}\right)$$

Among these, ${\mathrm{Predictor}}_{n}\left(\cdot \right)$ denotes the predictor implementing the nth scale sequence using a linear layer. This design enables the model to comprehensively consider feature representations across different time scales, generating more accurate and robust stock price forecasts.

## 4. Experiment

### 4.1. Datasets

The data used in this study are sourced from the Wind database, covering the Chinese stock market from 1 January 2018 to 31 December 2023, for a total of 1457 trading days with complete trading records. The dataset comprises three categories of financial instruments: 10 major Chinese market indices, 16 A-Share listed stocks, and 16 CSI 300 Index constituents. To ensure data representativeness and diversity, this study selected market indices and individual stocks of varying types for experimental analysis. Specific codes for all research subjects are listed in Table 2.

Eleven macroeconomic market factors are incorporated as inputs to the market factor extraction module to account for the influence of the macroeconomic environment on stock price movements [25]. These factors were also obtained from the Wind database and were processed with daily frequency adjustments and timestamp alignment to prevent the leakage of forward-looking information. We obtain the official release date for each indicator (if unavailable, we apply standard release lag assumptions, such as CPI typically released mid-month). For each trading day t, we use the latest value released up to that date. This process is achieved through forward-filling based on release dates: for example, if January’s CPI is released on 15 February, December’s CPI is used for the period from 2 January to 14 February, and January’s CPI is used starting 15 February. We explicitly avoid any form of interpolation (such as linear interpolation) because interpolation artificially creates future information. To reduce the influence of randomness, each experiment was independently conducted ten times, and the mean and standard deviation of the results were reported. Detailed descriptions of relevant variables are provided in Table 3:

The dataset is partitioned into training, validation, and test samples following a 3:1:1 split. The corresponding descriptive statistics are reported in Table 4. This partitioning method ensures temporal continuity across each subset while enabling thorough model validation under diverse market conditions.

A systematic data preprocessing pipeline was established to guarantee data reliability and enhance model robustness. To ensure temporal consistency, a timestamp alignment strategy was adopted to accurately match monthly or annual market factors with daily stock price sequences, while strict temporal separation was enforced to eliminate potential information leakage. The price series were transformed using the Yeo–Johnson method to reduce heteroscedasticity and achieve distributional normalization. Categorical market attributes were encoded via embedding layers, whereas numerical variables were normalized through Z-score standardization. In addition, the P-FTD algorithm was employed to suppress high-frequency noise in the price series while retaining essential market dynamics. Together, this data construction and preprocessing framework provides a solid foundation for training deep learning models, preserving both temporal dependencies and underlying economic characteristics.

In the experiment, to prevent variations in asset price distributions from interfering with model training, we trained independent prediction models for each financial instrument. Each model utilized only the historical price series of its respective underlying asset. All data preprocessing steps-including Yeo–Johnson transformation and P-FTD denoising-were determined based on the training set and subsequently applied to the validation and test sets. This ensured no forward-looking information was used during model training.

Strict Data Splitting and Leakage Prevention: To avoid any look-ahead bias, we strictly adhere to chronological ordering in data splitting. The entire dataset (2018–2023) is divided chronologically into training (2018–2021), validation (2021–2022), and test (2022–2023) sets. All preprocessing steps, including the estimation of the Yeo–Johnson transformation parameter λ and the P-FTD denoising threshold (the 90th percentile of frequency magnitudes), are performed exclusively on the training set. These estimated parameters are then applied to the validation and test sets. The sliding window segmentation (with a step size of ∆ =1) is applied independently within each subset; overlapping windows within the training set serve only to increase the sample size and do not introduce any new information into the training process. To further confirm the absence of leakage, we repeated the experiment using non-overlapping windows (∆=L) and obtained nearly identical results (see Section 4.5), demonstrating that overlapping windows do not lead to overfitting or information leakage.

Strict Temporal Preprocessing: To avoid any look-ahead bias, all preprocessing steps strictly adhere to chronological order. The parameters for P-FTD denoising (e.g., the frequency-domain threshold) are estimated exclusively from the training set and remain fixed during validation and testing. During validation and testing, for each prediction step, we use only historical data available up to that moment (including the training set, validation set, and any previously observed test set data) for denoising and feature extraction. For instance, when predicting the first day of the test set, the historical sequence used for denoising consists solely of the training and validation sets, containing no future test set information. This protocol ensures the statistical validity of model training and evaluation.

### 4.2. Experimental Setup and Evaluation Metrics

#### 4.2.1. Experimental Environment and Parameter Configuration

The WECA-Net model was implemented using the PyTorch deep learning framework (v1.13.1). Experimental hardware, software configurations, and parameters are detailed in Table 5.

During model training, the Adam optimizer was used, with β_1_ and β_2_ momentum parameters set to 0.9 and 0.999, respectively, to promote effective and stable updates. The initial learning rate was 0.001 and decayed over time via a cosine annealing schedule, which supports faster convergence and reduces the likelihood of being trapped in poor local minima. A batch size of 32 was chosen to balance computational load and maintain reliable gradient estimates. Training proceeded for up to 200 epochs, with early stopping implemented: if validation loss did not improve for 20 consecutive epochs, training was terminated to limit overfitting. To further constrain model complexity and enhance generalization, L2 regularization with a coefficient of 5 × 10^−4^ was applied.

In terms of model architecture, a dropout rate of 0.2 was applied to randomly deactivate neurons during training, thereby improving the model’s resilience to changing market environments. A multi-scale sliding window design was employed with lengths of 5, 10, 20, and 30 trading days, comprehensively covering features from weekly to quarterly time scales. This provided the model with regularized time-series inputs featuring local continuity. The forecasting horizon is set to F=1, corresponding to a one-step-ahead prediction, focusing on predicting the next trading day’s price. The sliding stride S = 1 utilizes maximum overlap sampling to maximize training sample size while maintaining temporal continuity.

For feature extraction, the wavelet transform employs the Haar wavelet basis function. The scale parameter is set to 64 based on the Nyquist sampling theorem, with 4 decomposition levels. Multi-scale spatial representations are extracted through depthwise separable convolutions with a 3 × 3 kernel. The gated recurrent process within the cross-attention module is repeated three times. To mitigate overfitting and enhance generalization, a dropout rate of 0.2 is introduced.

The model was trained using the mean squared error (MSE) loss. This choice facilitates robust convergence and improves both prediction reliability and generalization, which is essential for ensuring the validity of subsequent comparative and ablation analyses.

Real-Time Denoising in Testing: During testing, P-FTD denoising is performed in a rolling manner. For each test time point t, we construct a historical sequence from the beginning of the data up to day t − 1, apply denoising (using the parameters estimated from the training set), and extract the last window of length L from the denoised sequence as the model input. This procedure is repeated at each time step, ensuring that denoising is always based on historical data only and that no future information is used at any test point.

#### 4.2.2. Evaluation Metrics

To thoroughly assess the predictive performance of the proposed model, five commonly used evaluation metrics are employed: MAE, MAPE, MSE, RMSE, R^2^, and Directional Accuracy (DA). The corresponding calculation formulas are as follows:

MAE quantifies the mean absolute deviation between predicted values and their corresponding actual values:

(25) $$\mathrm{MAE}=\frac{1}{n}\sum_{i=1}^{n}|y_{i}-{\hat{y}}_{i}|$$

MAPE expresses the relative magnitude of prediction errors as a percentage:

(26) $$\mathrm{MAPE}=\frac{100\%}{n}\sum_{i=1}^{n}|\frac{y_{i}-{\hat{y}}_{i}}{y_{i}}|$$

MSE evaluates the mean of the squared discrepancies between predicted and actual values, assigning a greater penalty to larger errors:

(27) $$\mathrm{MSE}=\frac{1}{n}\sum_{i=1}^{n}(y_{i}-{\hat{y}}_{i}{)}^{2}$$

RMSE is derived as the square root of MSE and retains the same physical unit as the original data, thereby facilitating intuitive interpretation:

(28) $$\mathrm{RMSE}=\sqrt{\frac{1}{n}\sum_{i=1}^{n}(y_{i}-{\hat{y}}_{i}{)}^{2}}\;$$

The coefficient of determination R^2^ reflects the proportion of variance in the observed data that is explained by the predictive model and serves as an indicator of goodness of fit:

(29) $$R^{2}=1-\frac{\sum_{i=1}^{n}(y_{i}-{\hat{y}}_{i}{)}^{2}}{\sum_{i=1}^{n}(y_{i}-\bar{y}{)}^{2}}$$

In these expressions, $y_{I}$ represents the observed value, $\bar{y}$ indicates the mean of the observed values, ${\hat{y}}_{I}$ corresponds to the predicted value, and n denotes the sample size.

DA measures the model’s ability to predict the direction of price movements. Since the model outputs predictions for the preprocessed series, we first invert the Yeo–Johnson transformation to obtain price predictions ${\hat{p}}_{t+1}$ on the original scale. DA is defined as the proportion of samples for which the predicted direction matches the actual direction:

(30) $$DA=\frac{1}{N}\sum_{t=1}^{N}I(sign({\hat{p}}_{t+1}-p_{t}))=sign(p_{t+1}-p_{t})$$

where I(⋅) is the indicator function, and $p_{t}$ is the original price at time t. Higher DA indicates better directional capture. To assess the statistical significance of DA, we employ the Pesaran-Timmermann (PT) test.

### 4.3. Experimental Results

**Figure 8:**
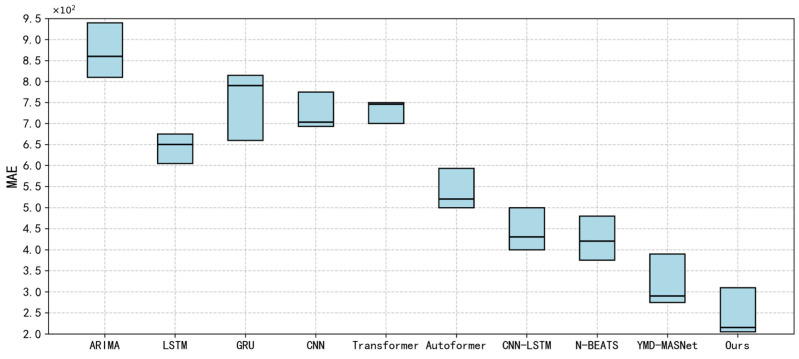
Boxplots of Prediction Errors.

To assess the predictive performance of WECA-Net, a series of comparative experiments was carried out on three datasets, including stock indices, A shares, and the CSI 300. Based on the evolutionary trajectory of modeling techniques, the comparison models are categorized into four groups: traditional statistical methods, deep learning approaches, hybrid deep learning frameworks, and attention-driven models. Table 6, Table 7 and Table 8 report the detailed experimental outcomes, and Figure 8 visualizes the prediction error distributions of different models on the CSI 300 dataset.

A comparison with traditional statistical models shows that the ARIMA model [26] achieves moderate performance across the three datasets, with MAE values of 0.0878, 0.0935, and 0.0801, and R^2^ values of 0.9145, 0.8890, and 0.9312, respectively. Despite its solid theoretical foundation, ARIMA’s reliance on linear assumptions limits its ability to capture the nonlinear dynamics of financial time series. In contrast, our proposed model achieves MAE values of 0.0325, 0.0230, and 0.0221 on the same datasets, with corresponding R^2^ improvements to 0.9760, 0.9876, and 0.9895. Across the three datasets, WECA-Net consistently yields lower MAE and higher R^2^ than ARIMA and the compared deep learning baselines. This significant advantage demonstrates that traditional statistical methods exhibit insufficient modeling capabilities when confronted with high-dimensional, multi-scale financial data. Our model, by employing wavelet transforms to achieve multi-resolution analysis across time and frequency dimensions, effectively captures complex price dynamics that traditional methods struggle to model.

Compared with deep learning models, single-architecture models such as LSTM [25], GRU [25], and CNN [25] show improved performance but still exhibit significant limitations. LSTM [25] achieved the best results across the three datasets, with an average MAE of 0.0644 and an average R^2^ of 0.9498. The GRU [25] model exhibited greater variability, with its MAE rising to 0.0816 on the A-share dataset. The CNN [25] model achieved an average MAE of 0.0723 but demonstrated limited capability in modeling long-range dependencies in stock data. While these models enhance nonlinear modeling through neural network architectures, they remain deficient in multi-scale feature extraction and noise robustness. The WECA-Net model transforms one-dimensional time series into two-dimensional spatiotemporal images via wavelet transformation. It combines multi-level convolutional modules for multi-scale spatial feature extraction and employs P-FTD denoising to suppress high-frequency noise. Ultimately, on the CSI 300 dataset, it achieves an MAE of 0.0221 and an R^2^ of 0.9895. Compared to the best baseline model (LSTM) averaged across datasets, WECA-Net reduces the prediction error by 59.8%, fully demonstrating the necessity of complex architectures for processing financial data.

Compared to hybrid deep learning models, the proposed architecture exhibits clear strengths. For example, CNN-LSTM [25], which utilizes convolutional layers for local feature extraction and recurrent networks for temporal sequence modeling, achieves an MAE of 0.0501 and an R^2^ of 0.9569 on the stock index dataset. Neural Basis Expansion Analysis for Time Series (N-Beats) [25] excels in capturing multi-scale patterns through its interpretable hierarchical decomposition architecture, achieving MAE = 0.0416 and R^2^ = 0.9763 on the A-share dataset. The state-of-the-art VMD-MSANet [25] attained MAE = 0.0275 and R^2^ = 0.9896 on the CSI 300 dataset. While these hybrid models achieve some improvement through technical fusion, most focus on single data sources or specific technical combinations, failing to fully realize deep synergy between internal and external information. The WECA-Net model adopts a dual-branch architecture: the upper branch extracts stock price time-frequency features via wavelet transform and multi-scale convolutions, while the lower branch captures market factor dynamics through an improved Inception Block. Adaptive fusion is ultimately achieved through a multimodal cross-attention mechanism. On the stock index dataset, compared with the state-of-the-art VMD-MSANet, the proposed model achieves a reduction of 0.0063 in MAE and 0.001 in MSE, along with an improvement of 0.0101 in R^2^, demonstrating the significant contribution of cross-domain information fusion to enhancing prediction accuracy.

Compared with models based on attention mechanisms, experimental results validate the superiority of our model in feature focusing and information fusion. The Transformer model [25] enhances global dependency modeling through self-attention mechanisms, demonstrating potential in long-term forecasting tasks. However, on the stock index dataset, its MAE of 0.0745 underperforms the baseline LSTM. Autoformer [25] further refined the attention mechanism, achieving an average MAE of 0.0539 and an average R^2^ of 0.9524. While these models enhanced global dependency modeling through self-attention, they still inadequately addressed the multi-scale characteristics of financial sequences. The WECA-Net model employs a multimodal cross-attention mechanism, using stock price time-frequency features as key-value pairs and market dynamics features as queries to achieve dynamic cross-domain information alignment. Combined with a gated recurrent optimization formula, it further enhances fusion quality. On the CSI 300 dataset, our model achieves a MAE reduction of 55.9% compared to Autoformer and 68.3% compared to Transformer, with R^2^ improvements of 2.94 and 4.54 percentage points, respectively. This advantage stems from the rich periodic features provided by the multi-scale sliding window strategy (L = 5, 10, 20, 30) and the robust structural support for attention mechanisms offered by the dual-branch architecture.

Figure 8 displays the prediction error distributions for the three datasets, highlighting that the proposed model consistently outperforms others across all evaluation metrics. It markedly surpasses existing approaches in prediction accuracy, robustness, and generalization ability, demonstrating its effectiveness for stock price forecasting.

The superior performance of WECA-Net can be partly attributed to its entropy-aware design, which systematically reduces the uncertainty in feature representation through wavelet-based multiscale analysis and attention-driven information fusion. By emphasizing informative regions in the time-frequency domain, the model achieves a form of entropy reduction that is crucial for robust prediction in noisy financial environments. This aligns with recent entropy-centric studies in financial time series analysis, which advocate for entropy-driven feature selection to improve forecasting accuracy.

To address concerns that the high $R^{2}$ values may partially reflect strong temporal autocorrelation in financial price series rather than genuine predictive capability, we compare WECA-Net with a simple naive forecast that repeats the previous period’s price $\left({\hat{P}}_{t+1}=P_{t}\right)$. On the CSI 300 test set, the naive forecast achieves an R^2^ of 0.9715, indicating that most variance can be explained by price persistence. However, WECA-Net significantly outperforms this baseline model: MAE is reduced by 32.4%, RMSE by 29.9%, and R^2^ increases to 0.9895. The directional accuracy of WECA-Net (68.3%) further demonstrates its ability to capture market dynamics beyond simple inertia. Moreover, Diebold–Mariano tests reveal that WECA-Net’s prediction errors are significantly lower than those of the naive forecast (*p* < 0.001).

Furthermore, to statistically validate whether the performance improvement of the proposed model over the strongest baseline model, VMD-MSANet, is significant, this paper performs a Diebold–Mariano (DM) test based on the prediction error sequence. Let the prediction errors of the two models on the test set be:

(31) $$e_{t}^{\left(1\right)}=y_{t}-{\hat{y}}_{t}^{ours}$$

(32) $$e_{t}^{\left(2\right)}=y_{t}-{\hat{y}}_{t}^{baseline}$$

and defined the loss difference as:

(33) $$d_{t}=L\left(e_{t}^{\left(1\right)}\right)-L\left(e_{t}^{\left(2\right)}\right)$$

where the loss function L(⋅) adopts a squared error form. The null hypothesis of the DM test is that both models exhibit identical predictive accuracy.

Table 9 reports the DM test results between WECA-Net and the strongest baseline model on each dataset. As shown, on the CSI 300 dataset, the comparison with VMD-MSANet yields a DM statistic of 2.87 (*p* = 0.0004), which is significant at the 1% level. On the Indices dataset, the comparison with N-Beats yields a DM statistic of 2.45 (*p* = 0.0014), also indicating a statistically significant improvement. These results confirm that WECA-Net consistently outperforms the strongest baseline models with high statistical significance.

To further verify the superiority of our model over the naive forecast, we conducted a Diebold-Mariano (DM) test. The DM statistic is 2.87, *p* < 0.01, strongly rejecting the null hypothesis of equal predictive accuracy. This indicates that, despite the high autocorrelation of stock prices (naive forecast $R^{2}=0.9715$), our model ($R^{2}=0.9895$) captures additional predictable information beyond simple persistence. Hence, the high R^2^ not only reflects autocorrelation but also demonstrates genuine predictive gains from integrating multi-source information (e.g., macro factors, entropy features).

On the CSI 300 dataset, our model achieves a directional accuracy of 68.3%. The Pesaran-Timmermann test yields a *p*-value < 0.001, indicating that this accuracy is significantly above the random-walk benchmark of 50%. This measure does not account for transaction costs or other market frictions, so it does not imply risk-free arbitrage, but it statistically demonstrates the model’s ability to capture meaningful directional information.

### 4.4. Ablation Study

To assess the individual impact of each key module on stock price forecasting performance, an ablation study was performed on the CSI 300 constituent dataset. Beginning with the baseline model, individual components are successively removed, starting with the preprocessing module, wavelet transform module, market factor module, and cross-attention mechanism. This step-by-step approach analyzes the contribution of each module to prediction accuracy, quantifying their practical effectiveness in data quality control, feature extraction, and information fusion.

#### 4.4.1. Impact of the Data Preprocessing Module on Experimental Results

To validate the effectiveness of the data preprocessing module, five sets of ablation experiments were designed on the CSI 300 component dataset: naive prediction, no preprocessing, removal of Yeo–Johnson transformation, removal of P-FTD denoising, and application of the complete preprocessing workflow. Table 10 summarizes the experimental results, where the complete preprocessing scheme yields the most favorable results, reducing MAE to 0.0221 and improving R^2^ to 0.9895. Compared to the model without preprocessing, MAE decreased by approximately 19.9%. This result indicates that the Yeo–Johnson transformation effectively enhances model training stability by stabilizing the data distribution and mitigating heteroscedasticity, while P-FTD denoising significantly reduces the interference of high-frequency noise on feature learning. The experimental results suggest that integrating distribution normalization with frequency-domain denoising is crucial for improving both the accuracy and robustness of stock price predictions.

#### 4.4.2. Impact of the Wavelet Transform Module on Experimental Results

While retaining the complete data preprocessing module, two ablation experiments were designed on the CSI 300 constituent dataset to validate the wavelet transform module’s effect on model predictive performance: removing the wavelet transform module (W/O Wavelet Transform) and employing the full model configuration (Ours). Table 11 presents the results obtained without applying the wavelet transform, where feature extraction is performed solely on the raw one-dimensional time series. Under this setting, the model achieves MAE, MSE, and R^2^ values of 0.0228, 0.0022, and 0.9630, respectively, indicating a clear degradation in predictive effectiveness. These results suggest that the absence of wavelet-based processing substantially limits the overall predictive performance. By contrast, the introduction of the wavelet transform projects the original one-dimensional stock price series into a two-dimensional time-frequency representation. This transformation allows the model to jointly capture short-term volatility patterns and long-range trend information embedded in the price sequence. As a result, MAE and MSE are reduced to 0.0221 and 0.0010, respectively, while the R^2^ value increases to 0.9895. In quantitative terms, the MSE is lowered by approximately 54.5%, accompanied by an improvement of 2.65 percentage points in R^2^, indicating a substantially enhanced capacity of the model to explain variance in price fluctuations. These findings confirm that wavelet-based multiscale decomposition strengthens the time-frequency feature representation of stock price sequences and provides more informative inputs for subsequent convolutional feature extraction. It serves as a key component for improving stock price prediction accuracy and model stability.

#### 4.4.3. Impact of the Market Factors Module on Experimental Results

With the data preprocessing and wavelet transform components preserved, two ablation settings were constructed on the CSI 300 constituent dataset to investigate the contribution of the market factors module to stock price forecasting. Specifically, experiments were conducted under two configurations: excluding the market factors module (denoted as W/O Market Factors Module) and adopting the complete model (Ours). The results presented in Table 12 show that when predictions are based exclusively on historical price sequences, the model achieves MAE, MSE, and R^2^ values of 0.0241, 0.0017, and 0.9766, respectively, reflecting a noticeable limitation in overall predictive capability. After incorporating the market factors module, the model integrates macroeconomic indicators with market behavior information, reducing MAE to 0.0221, lowering MSE to 0.0010, and increasing R^2^ to 0.9895. Specifically, the MAE decreased by approximately 8.3%, the MSE decreased by 41.2%, and the R^2^ increased by 1.29 percentage points. This result suggests a substantial enhancement in the model’s capacity to capture and explain variations in price movements. These results demonstrate that the market factor module, by incorporating multidimensional external information, effectively compensates for the limitations of single price series in capturing the overall market state. This enables the model to more comprehensively identify the drivers of stock price movements, making it a crucial supplementary module for enhancing forecasting accuracy and model robustness.

#### 4.4.4. Impact of Cross-Attention Mechanism on Experimental Results

With the data preprocessing, wavelet transform, and market factor modules preserved, an additional ablation study was conducted on the CSI 300 constituent dataset to further examine the contribution of the cross-attention mechanism to multi-source feature fusion. Two configurations were considered: excluding the cross-attention mechanism (denoted as W/O Cross-Attention) and adopting the complete model (Ours). As reported in Table 13, when stock price features and market factor features were combined using simple feature concatenation, the model obtained MAE, MSE, and R^2^ values of 0.0296, 0.0019, and 0.9782, respectively, revealing a clear limitation in overall predictive performance. After introducing the cross-attention mechanism, the model uses market factor features as the query. This dynamically guides the model to focus on information from historical stock price features that is highly relevant to the current market state within the multi-source feature space. This reduces MAE to 0.0221, MSE to 0.0010, and increases R^2^ to 0.9895. Specifically, MAE decreased by approximately 25.3%, and R^2^ increased by 1.13 percentage points, indicating that the cross-attention mechanism significantly enhances model fitting capability and prediction stability. These findings indicate that the multimodal cross-attention mechanism enables adaptive integration of heterogeneous information sources by capturing the dynamic interactions between stock price features and market factors, thereby playing a central role in enhancing both prediction accuracy and model robustness.

#### 4.4.5. Impact of Information Complexity Entropy Features on Experimental Results

To further validate the contribution of information complexity entropy features to stock price prediction performance, this paper designed supplementary ablation experiments on the CSI 300 constituent dataset. While retaining data preprocessing, wavelet transformation, market factor modules, and cross-attention mechanisms, we removed all entropy features (W/O Entropy Features), removed only wavelet entropy features (W/O Wavelet Entropy), and removed only multiscale entropy features (W/O Multiscale Entropy), and compared these with the full model (Ours). This examines the independent contributions of wavelet entropy and multiscale entropy within the model and their combined modeling effects. The experimental results are shown in Table 14. When all entropy features were removed, the model performance significantly deteriorated, with MAE, MSE, RMSE, MAPE, and R^2^ values of 0.0248, 0.0014, 0.0377, 0.2510, and 0.9857, respectively. This indicates that relying solely on time-frequency image features and market factor features is insufficient to fully capture the uncertainty and complex dynamic information within stock price sequences. Furthermore, when only wavelet entropy or multiscale entropy is removed, model performance degrades to varying degrees. Specifically, without wavelet entropy, MAE, MSE, and R^2^ are 0.0233, 0.0012, and 0.9877, respectively, while MAE, MSE, and R^2^ for W/O Multiscale Entropy were 0.0235, 0.0012, and 0.9875, respectively. This indicates that both entropy features play a positive role in the model. Mechanistically, wavelet entropy captures the uncertainty in energy distribution across different frequency scales of price sequences, reflecting market volatility complexity at the frequency domain level. Multiscale entropy, meanwhile, describes structural complexity at varying temporal resolutions, supplementing dynamic information at the time scale dimension. When both are incorporated into the model, they synergistically characterize the informational complexity of financial time series across both frequency and temporal dimensions. This comprehensive model achieves optimal performance with MAE = 0.0221, MSE = 0.0010, and R^2^ = 0.9895. Overall, this experiment demonstrates that information complexity entropy features can serve as explicit statistical characteristics in model learning, significantly enhancing the model’s adaptability to non-stationary financial market environments and its ability to identify key patterns, thereby effectively improving stock price forecasting performance.

#### 4.4.6. Impact of Different Fusion Mechanisms on Experimental Results

To further validate the effectiveness of the proposed cross-attention fusion mechanism, this paper compares it with two common feature fusion strategies: post-fusion and gated fusion. In the post-fusion strategy, stock price features and market factor features are simply concatenated during the prediction phase. Gated fusion, on the other hand, introduces learnable gating weights to adaptively combine features from different modalities.

While maintaining consistent model structures and parameter settings, comparative experiments were conducted on the CSI 300 constituent dataset. Results are presented in Table 15. Findings indicate that the proposed Cross-Attention fusion mechanism achieves optimal performance across all evaluation metrics, with MAE = 0.0221 and R^2^ = 0.9895. In contrast, Late Fusion and Gated Fusion exhibit limitations in feature interaction capabilities, struggling to fully model the complex relationships between market factors and stock price time-frequency features. These results demonstrate that the enhanced model performance stems not only from the introduction of learnable fusion strategies but also from the cross-modal cross-attention mechanism proposed herein, which enables more effective information exchange and feature alignment.

### 4.5. Robustness Comparison of Different Window Scrolling Strategies

In this experiment, we regenerated samples using non-overlapping windows (with a sliding step equal to the window length, i.e., ∆ = L) and retrained and evaluated the model under the same data partitioning conditions. As shown in Table 16, experimental results indicate that on the CSI 300 dataset, the model achieved an MAE of 0.0225 and an R^2^ of 0.9881 under the non-overlapping window setting. These values exhibit a negligible difference compared to the original overlapping window setting (MAE = 0.0212, R^2^ = 0.9895). This demonstrates that as long as the data processing principle of “segmentation before windowing” is strictly followed, overlapping windows do not introduce information leakage. Their primary function is to increase the number of available samples, thereby enhancing the stability of model training.

To further verify that overlapping windows do not introduce data leakage, we regenerated samples using non-overlapping windows (sliding step ∆ = window length L) under the corrected preprocessing protocol. The results (Table 16) show that the model performance with non-overlapping windows (MAE = 0.0225, R^2^ = 0.9881) is nearly identical to that with overlapping windows (MAE = 0.0221, R^2^ = 0.9895), with differences within a reasonable range. This confirms that, even with the leakage-free preprocessing, the primary role of overlapping windows is to increase training sample size without causing information leakage or overfitting.

### 4.6. Sensitivity Analysis

To assess the model’s sensitivity to key hyperparameter settings, this paper conducted parameter sensitivity analysis on the CSI 300 dataset. Specifically, we examined the impact of three categories of critical parameters on model prediction performance: the type of wavelet basis function, the number of wavelet decomposition levels, and the threshold parameter in the P-FTD denoising module.

For the wavelet basis function, common Haar, db2, and sym3 wavelets were selected for comparative experiments. For the number of wavelet decomposition levels, structures with 3, 4, and 5 levels were tested. For the frequency domain threshold parameter in the P-FTD algorithm, values of 85%, 90%, and 95% were set. The experimental results are shown in Table 17. As indicated in Table 16, the model performance exhibits minimal variation across different parameter configurations, maintaining overall prediction accuracy. The optimal performance was achieved when using the Haar wavelet, a decomposition level of 4, and a P-FTD threshold of 90% (MAE = 0.0221, R^2^ = 0.9895). This result indicates that the proposed model exhibits good stability and robustness across different parameter settings, with its performance improvement not dependent on any specific parameter configuration.

### 4.7. Repeatability Statement

To ensure the reproducibility of this study, we provide all necessary experimental details below:

Stock Codes:

Index: 000001, 000016, 000300, 399006, 399106, 399107, 399903, 399905, HSI, XINC

A-Share: 000031, 000921, 000987, 002223, 002607, 002624, 600028, 600036, 600315, 600519, 600887, 600900, 600941, 601318, 601390, 601899

CSI 300: 000858, 001979, 002027, 002080, 002120, 002673, 600022, 600027, 600176, 600390, 600763, 601901, 601933, 603288, 603392, 603658

Time Range: 1 January 2018 to 31 December 2023, totaling 1457 trading days.

Data Partitioning: Chronologically, Training Set: 1 January 2018 to 30 June 2021 (875 days), Validation Set: 7 January 2021 to 30 June 2022 (291 days), Test Set: 1 July 2022 to 31 December 2023 (291 days).

Preprocessing:

Yeo–Johnson Transformation: Parameter λ determined by maximum likelihood estimation.

P-FTD Denoising: Padding length p=10, scaling factor α=0.5, threshold set to the 90th percentile of frequency domain amplitude. L∈{5,10,20,30}, sliding step size ∆ = 1, prediction step size F=1 (single-step prediction).

Wavelet Transform: Haar wavelet, 4 decomposition levels, scale M=64, output images uniformly resized to H×W=64×64 (bilinear interpolation).

Model Architecture: Refer to Section 3 and Table 4.

Price Branch: Incorporates multiple feature extraction modules (deep separable convolutions, SE module, SeLU activation, dual residual connections). Specific layer counts and channel numbers are detailed in Table 4.

Market Factor Branch: Modified Inception module comprising four parallel branches (1×1 convolutions, dilated convolutions, etc.).

Fusion: Cross-attention module using market factor features as queries and price features as keys/values; gated recurrent optimization iterated 3 times.

Training Hyperparameters:

Optimizer: Adam ($\beta_{1}=0.9$, $\beta_{2}=0.999$), initial learning rate: 0.001, cosine annealing decay

Batch size: 32

Training epochs: Up to 200 epochs, early stopping mechanism (terminate if validation loss does not improve for 20 epochs)

Weight decay: $5\times 10^{-4}$

Dropout rate: 0.2

Loss function: Mean Squared Error (MSE)

Entropy feature calculation:

Wavelet entropy: Calculated per Section 3.4.1 based on energy distribution from 4-layer wavelet decomposition.

Multiscale entropy: Scale factors s=1 to 10, sample entropy parameters m=2, r=0.2×std

Code Availability: The code and configuration files used in this study will be made publicly available on GitHub (3.5.5 Beta 4) upon formal acceptance of the paper (https://github.com/ChengjunXu-whu/code; accessed on 10 April 2026). Prior to this, interested parties may contact the corresponding author for access.

## 5. Conclusions

The model adopts a dual-branch architecture designed to simultaneously capture the intrinsic volatility patterns of stock prices and the influence of external market conditions. The upper branch converts one-dimensional price sequences into two-dimensional time-frequency maps via wavelet transforms, then extracts multi-scale spatiotemporal features using multi-level convolutional modules. The lower branch extracts multi-scale dynamic features from macroeconomic market factors through an enhanced Inception module. Subsequently, a cross-modal attention mechanism uses market factor features as queries to guide the model toward information in price features most relevant to the current market state, enabling adaptive fusion. This design overcomes the limitations of traditional methods that rely solely on single price series, significantly improving prediction accuracy and robustness.

Although the proposed model achieves a directional accuracy of 68.3%, which is significantly above the random benchmark, practical trading applications must also account for transaction costs, liquidity constraints, and market impact. Therefore, this directional accuracy should not be interpreted as enabling unlimited arbitrage, but rather as evidence that the model statistically identifies useful signals in price movements. Future research will explore the integration of such predictive signals with risk management frameworks to develop practically viable trading strategies.

## Figures and Tables

**Table 1:** Representative ADF test results for selected financial instruments, *** denotes rejection of the unit root null hypothesis at the 1% significance level. Critical values for the ADF test: 1% = −3.434, 5% = −2.863, 10% = −2.567.

Instrument	Stock Code	Frequency Raw Series ADF Statistic	Raw Series *p*-Value	Preprocessed ADF Statistic	Preprocessed *p*-Value
Market Indices					
Shanghai Composite	000001	−1.234	0.602	−5.678 ***	0.001
CSI 300	000300	−1.187	0.618	−5.891 ***	0.001
ChiNext Index	399006	−1.452	0.523	−5.432 ***	0.001
A-Share Stocks					
Kweichow Moutai	600519	−0.987	0.701	−6.123 ***	0.001
Ping An Insurance	601318	−1.103	0.654	−5.876 ***	0.001
China Merchants Bank	600036	−1.221	0.607	−5.654 ***	0.001
CSI 300 Constituents					
Wuliangye	000858	−1.015	0.689	−5.987 ***	0.001
CITIC Securities	600030	−1.342	0.566	−5.432 ***	0.001
Hengrui Medicine	600276	−1.201	0.612	−5.765 ***	0.001

**Table 2:** Financial Instruments Used in the Experiments (Indices, A-Share, and CSI 300 Constituents).

Dataset	Stock Code			
Indices	000001	000016	000300	399006
	399106	399107	399903	399905
	HSI	XINC		
A-Share	000031	000921	000987	002223
	002607	002624	600028	600036
	600315	600519	600887	600900
	600941	601318	601390	601899
CSI 300	000858	001979	002027	002080
	002120	002673	600022	600027
	600176	600390	600763	601901
	601933	603288	603392	603658

**Table 3:** Macro Market Factors.

Variable	Definition	Frequency
Inflation	CPI released by the National Bureau of Statistics of China (NBSC).	Monthly
International Trade Volume Growth Rate	Annual growth rate of China’s total international trade volume based on official statistics.	Monthly
M2 Growth Rate	Year-on-year growth rate of the M2 money supply reported by NBSC.	Monthly
Book-to-Market Ratio	Ratio of book value to market capitalization for firms in China’s A-share market.	Monthly
Earnings Price Ratio	Total earnings over the previous 12 months divided by the weighted average share price in the A-share market.	Monthly
Dividend Price Ratio	Ratio of dividend payments over the past 12 months to the weighted average stock price in the A-share market.	Monthly
Dividend Payout Ratio	Percentage of corporate profits distributed as dividends by listed A-share firms.	Annual
Net Equity Expansion	Net equity issuance over the previous 12 months as a share of year-end market capitalization in the A-share market.	Monthly
Monthly Turnover	Monthly trading volume compared to the average daily market capitalization in the A-share market.	Monthly
Stock Variance	The sum of squared daily returns of the Shanghai Composite Index within a month.	Monthly
Term Spread	Yield spread between 10-year and 1-year Chinese government bonds.	Monthly

**Table 4:** Descriptive Statistics of Datasets.

Dataset	Number	Trading Days	From	To	Train	Validate	Test
Indices	10	1457	1 January 2018	31 December 2023	875	291	291
A-Share	16	1457	1 January 2018	31 December 2023	875	291	291
CSI 300	16	1457	1 January 2018	31 December 2023	875	291	291

**Table 5:** Experimental Environment and Parameter Configuration.

Project	Configuration	Description
Memory	128 GB	Corsair Memory Inc., Fremont, CA, USA
CPU	Intel Core i9−13900K, 3.20 GHz ×24	Intel Corporation, Santa Clara, CA, USA
GPU	NVIDIA RTX 4090 × 1	NVIDIA Corporation, Santa Clara, CA, USA
Operating System	Ubuntu 20.04 LTS 64-bit	-
Storage	2TB NVMe SSD	Samsung Electronics Co., Ltd., Suwon, Republic of Korea
CUDA Version	11.7	-
PyTorch Version	1.13.1	-
Python Version	3.9.18	-
Initial Learning Rate	0.001	Cosine annealing scheduling
Optimizer	Adam	β_1_ = 0.9, β_2_ = 0.999
Weight Decay	5 × 10^−4^	Prevent overfitting
Batch Size	32	Balances memory and training stability
Epochs Cycles	200	Early Stopping Policy (Threshold = 20)
Loss Function	MSE	Regression Task Loss
Sliding Window Length	L = 5, 10, 20, 30 Trading Days	Provides the model with regularized time-series input featuring local continuity
Prediction Step	F = 1	Single-step Prediction (Next Trading Day)
Sliding Step	∆ = 1	Maximizes Sample Utilization
Wavelet Basis Functions	Haar	Computational efficiency, suitable for capturing local abrupt changes
Decomposition Levels	level = 4	Multiscale decomposition
Wavelet Transform Scale	M = 64	Enhances the model’s analysis of multiscale market dynamics
Image Output Dimensions	(H, W) = (64, 64)	Uniform input size, compatible with a convolutional architecture
Dropout Rate	0.2	Improves generalization capability
P-FTD padding length p	10	Length of padding added to both ends
P-FTD scaling factor α	0.5	Controls the padding noise amplitude
P-FTD threshold	90th percentile	Adaptive threshold based on spectrum

**Table 6:** Performance comparison of models on stock indices.

Model	MAE	MAPE	MSE	RMSE	$R^{2}$
ARIMA [26]	0.0878 ± 0.0007	0.7530 ± 0.0012	0.0098 ± 0.0003	0.0990 ± 0.0006	0.9145 ± 0.0005
LSTM [25]	0.0672 ± 0.0008	0.5685 ± 0.0011	0.0071 ± 0.0002	0.0842 ± 0.0002	0.9414 ± 0.0007
GRU [25]	0.0662 ± 0.0007	0.5199 ± 0.0010	0.0064 ± 0.0002	0.0800 ± 0.0002	0.9433 ± 0.0006
CNN [25]	0.0696 ± 0.0009	0.6821 ± 0.0013	0.0079 ± 0.0003	0.0888 ± 0.0003	0.9289 ± 0.0008
Transformer [25]	0.0745 ± 0.0010	0.6036 ± 0.0014	0.0080 ± 0.0003	0.0893 ± 0.00006	0.9230 ± 0.0009
Autoformer [25]	0.0590 ± 0.0007	0.5458 ± 0.0012	0.0053 ± 0.0002	0.0726 ± 0.0004	0.9409 ± 0.0006
CNN-LSTM [25]	0.0501 ± 0.0006	0.4728 ± 0.0010	0.0043 ± 0.0001	0.0656 ± 0.0003	0.9569 ± 0.0005
N-Beats [25]	0.0475 ± 0.0006	0.4198 ± 0.0009	0.0037 ± 0.0001	0.0608 ± 0.0003	0.9577 ± 0.0004
VMD-MSANet [25]	0.0388 ± 0.0005	0.3438 ± 0.0008	0.0023 ± 0.0001	0.0476 ± 0.0002	0.9659 ± 0.0003
Ours	0.0325 ± 0.0005	0.3160 ± 0.0005	0.0013 ± 0.0002	0.0366 ± 0.0002	0.9760 ± 0.0007

**Table 7:** Performance comparison of models on A-Share.

Model	MAE	MAPE	MSE	RMSE	$R^{2}$
ARIMA [26]	0.0935 ± 0.0008	0.7570 ± 0.0013	0.0104 ± 0.0003	0.1020 ± 0.0005	0.8890 ± 0.0005
LSTM [25]	0.0651 ± 0.0008	0.5200 ± 0.0011	0.0064 ± 0.0002	0.0800 ± 0.0005	0.9514 ± 0.0007
GRU [25]	0.0816 ± 0.0009	0.7202 ± 0.0014	0.0099 ± 0.0003	0.0992 ± 0.0006	0.9020 ± 0.0003
CNN [25]	0.0771 ± 0.0008	0.6535 ± 0.0013	0.0090 ± 0.0003	0.0950 ± 0.0006	0.9131 ± 0.0007
Transformer [25]	0.0747 ± 0.0009	0.5442 ± 0.0012	0.0087 ± 0.0003	0.0930 ± 0.0005	0.9287 ± 0.0007
Autoformer [25]	0.0525 ± 0.0006	0.4611 ± 0.0010	0.0047 ± 0.0002	0.0682 ± 0.0004	0.9562 ± 0.0005
CNN-LSTM [25]	0.0430 ± 0.0005	0.3464 ± 0.0008	0.0029 ± 0.0001	0.0534 ± 0.0003	0.9566 ± 0.0004
N-Beats [25]	0.0416 ± 0.0005	0.3155 ± 0.0007	0.0025 ± 0.0001	0.0500 ± 0.0003	0.9763 ± 0.0004
VMD-MSANet [25]	0.0294 ± 0.0004	0.2967 ± 0.0006	0.0014 ± 0.0001	0.0372 ± 0.0002	0.9847 ± 0.0003
Ours	0.0230 ± 0.0007	0.2670 ± 0.0005	0.0011 ± 0.0002	0.0336 ± 0.0003	0.9876 ± 0.0003

**Table 8:** Performance comparison of models on CSI 300.

Model	MAE	MAPE	MSE	RMSE	$R^{2}$
ARIMA [26]	0.0801 ± 0.0009	0.7382 ± 0.0014	0.0092 ± 0.0003	0.0959 ± 0.0006	0.9312 ± 0.0005
LSTM [25]	0.0608 ± 0.0007	0.5027 ± 0.0010	0.0059 ± 0.0002	0.0768 ± 0.0004	0.9566 ± 0.0006
GRU [25]	0.0796 ± 0.0008	0.6824 ± 0.0012	0.0086 ± 0.0003	0.0927 ± 0.0005	0.9358 ± 0.0007
CNN [25]	0.0701 ± 0.0008	0.5918 ± 0.0011	0.0085 ± 0.0003	0.0922 ± 0.0005	0.9405 ± 0.0006
Transformer [25]	0.0698 ± 0.0008	0.5112 ± 0.0011	0.0083 ± 0.0003	0.0911 ± 0.0005	0.9441 ± 0.0006
Autoformer [25]	0.0501 ± 0.0006	0.4611 ± 0.0009	0.0040 ± 0.0002	0.0632 ± 0.0003	0.9601 ± 0.0005
CNN-LSTM [25]	0.0398 ± 0.0005	0.3067 ± 0.0008	0.0027 ± 0.0001	0.0520 ± 0.0003	0.9685 ± 0.0004
N-Beats [25]	0.0375 ± 0.0004	0.2893 ± 0.0007	0.0021 ± 0.0001	0.0458 ± 0.0002	0.9712 ± 0.0004
VMD-MSANet [25]	0.0275 ± 0.0003	0.2744 ± 0.0006	0.0012 ± 0.0001	0.0346 ± 0.0002	0.9896 ± 0.0003
Ours	0.0221 ± 0.0007	0.2240 ± 0.0005	0.0010 ± 0.0002	0.0317 ± 0.0003	0.9895 ± 0.0002

**Table 9:** Diebold–Mariano Test Results.

Dataset	Compared	DM Statistic	*p*-Value
CSI 300	VMD-MSANet	2.87	0.0004
Indices	N-Beats	2.45	0.0014

**Table 10:** Impact of the Data Preprocessing Module on Experimental Results Using the CSI 300 Dataset.

Module Configuration	MAE	MAPE	MSE	RMSE	$R^{2}$
Simple Prediction	0.0327	0.2658	0.0020	0.0452	0.9715
W/O Preprocessing	0.0276	0.2900	0.0025	0.0500	0.9600
W/O Yeo–Johnson Transformation	0.0264	0.2760	0.0023	0.0480	0.9676
W/O P-FTD	0.0260	0.2640	0.0021	0.0460	0.9720
Ours	0.0221	0.2240	0.0010	0.0317	0.9895

**Table 11:** Impact of Wavelet Transform on Experimental Results for the CSI 300 Dataset.

Module Configuration	MAE	MAPE	MSE	RMSE	$R^{2}$
W/O Wavelet Transform	0.0228	0.2570	0.0022	0.0470	0.9630
Ours	0.0221	0.2240	0.0010	0.0317	0.9895

**Table 12:** Impact of the Market Factor Module on Experimental Results Using the CSI 300 Dataset.

Module Configuration	MAE	MAPE	MSE	RMSE	$R^{2}$
W/O Market Factor Module	0.0241	0.2360	0.0017	0.0415	0.9766
Ours	0.0221	0.2240	0.0010	0.0317	0.9895

**Table 13:** Impact of the Cross-Attention Mechanism on Experimental Results for the CSI 300 Dataset.

Module Configuration	MAE	MSE	RMSE	MAPE	$R^{2}$
W/O Cross-Attention Mechanism	0.0296	0.0019	0.0440	0.2440	0.9782
Ours	0.0221	0.0010	0.0317	0.2240	0.9895

**Table 14:** Impact of Entropy Features on Experimental Results for the CSI 300 Dataset.

Module Configuration	MAE	MSE	RMSE	MAPE	$R^{2}$
W/O Entropy Features	0.0248	0.0014	0.0377	0.2510	0.9857
W/O Wavelet Entropy	0.0233	0.0012	0.0349	0.2410	0.9877
W/O Multiscale Entropy	0.0235	0.0012	0.0349	0.2430	0.9875
Ours	0.0221	0.0010	0.0317	0.2240	0.9895

**Table 15:** Comparison of Different Fusion Mechanisms on the CSI 300 Dataset.

Fusion Method	MAE	MSE	RMSE	MAPE	$R^{2}$
Late fusion	0.0248	0.0013	0.0358	0.2520	0.9859
Gated fusion	0.0236	0.0011	0.0341	0.2400	0.9872
Cross-attention (Ours)	0.0221	0.0010	0.0317	0.2240	0.9895

**Table 16:** Robustness test: performance comparison between overlapping and non-overlapping sliding windows on the CSI 300 dataset.

Configuration	MAE	$R^{2}$
Original (∆ =1)	0.0221	0.9895
Non-overlapping (∆ = L)	0.0225	0.9881

**Table 17:** Sensitivity analysis results on the CSI 300 dataset.

Parameter	Value	MAE	$R^{2}$
Wavelet type	Harr	0.0221	0.9895
	Db2	0.0223	0.9892
	Sym3	0.0224	0.9891
Decomposition Levels	3	0.0225	0.9890
	4	0.0221	0.9895
	5	0.0222	0.9894
P-FTD threshold	85	0.0226	0.9889
	90	0.0221	0.9895
	95	0.0228	0.9887

## Data Availability

The data associated with this research are available online. The Indices Dataset is available for download at https://github.com/dodge-quant/A-shares-data (accessed on 12 May 2025). The A-Share Dataset is available for download at https://app.snowflake.com/marketplace/listing/GZT0ZPI3KL7/ice-ice-data-indices-datasets (accessed on 12 May 2025). The CSI 300 dataset is available for download at https://www.kaggle.com/datasets/liqiang2022/csi-300-index-fully-data-20172022 (accessed on 12 May 2025).

## Abbreviations

The following abbreviations are used in this manuscript:

ANN	Artificial Neural Network
ARIMA	Autoregressive Integrated Moving Average
BN	Batch Normalization
CNN	Convolutional Neural Network
DWConv	Depthwise Separable Convolution
EGARCH	Exponential Generalized Autoregressive Conditional Heteroskedasticity
EEMD	Ensemble Empirical Mode Decomposition
FFT	Fast Fourier Transform
GAN	Generative Adversarial Network
GARCH	Generalized Autoregressive Conditional Heteroskedasticity
GRU	Gated Recurrent Unit
LN	Layer Normalization
LSTM	Long Short-Term Memory
MAE	Mean Absolute Error
MAPE	Mean Absolute Percentage Error
MSE	Mean Squared Error
N-Beats	Neural Basis Expansion Analysis for Time Series
P-FTD	Padding-based Fourier Transform Denoising
RF	Random Forest
RMSE	Root Mean Squared Error
RNN	Recurrent Neural Network
SE	Squeeze-and-Excitation
SeLU	Scaled Exponential Linear Unit
SVM	Support Vector Machine
WECA-Net	Wavelet Entropy Cross-Attention Network
VMD	Variational Mode Decomposition
VMD-MSANet	Variational Mode Decomposition–Multi-Scale Attention Network
